# Elevated Contribution of Low Nucleic Acid Prokaryotes and Viral Lysis to the Prokaryotic Community Along the Nutrient Gradient From an Estuary to Open Ocean Transect

**DOI:** 10.3389/fmicb.2020.612053

**Published:** 2020-12-15

**Authors:** Chen Hu, Xiaowei Chen, Liuqian Yu, Dapeng Xu, Nianzhi Jiao

**Affiliations:** ^1^State Key Laboratory of Marine Environmental Science, College of Ocean and Earth Sciences, Institute of Marine Microbes and Ecospheres, Xiamen University, Xiamen, China; ^2^Fujian Key Laboratory of Marine Carbon Sequestration, Xiamen University, Xiamen, China; ^3^Department of Ocean Science, The Hong Kong University of Science and Technology, Hong Kong, China

**Keywords:** HNA and LNA prokaryotes, protozoan grazing, viral lysis, nutrient gradient, northern South China Sea

## Abstract

Prokaryotes represent the largest living biomass reservoir in aquatic environments and play a crucial role in the global ocean. However, the factors that shape the abundance and potential growth rate of the ecologically distinct prokaryotic subgroups [i.e., high nucleic acid (HNA) and low nucleic acid (LNA) cells] along varying trophic conditions in the ocean remain poorly understood. This study conducted a series of modified dilution experiments to investigate how the abundance and potential growth rate of HNA and LNA prokaryotes and their regulating factors (i.e., protozoan grazing and viral lysis) change along a cross-shore nutrient gradient in the northern South China Sea. The results showed that the abundance of both HNA and LNA cells was significantly positively correlated with the abundance of heterotrophic nanoflagellates and viruses, whereas only HNA abundance exhibited a significant positive correlation with nutrient level. With a decreasing nutrient concentration, the potential growth rate of the HNA subgroup declined significantly, while that of the LNA subgroup was significantly enhanced, leading to an elevated relative potential growth rate of the LNA to HNA subgroup under decreasing nutrient levels. Furthermore, our data revealed different regulatory roles of protozoan grazing and viral lysis on the HNA and LNA subgroups, with HNA suffering higher mortality pressure from grazing than from lysis in contrast to LNA, which experienced equivalent pressures. As the nutrient levels declined, the relative contribution of lysis to the mortality of the HNA subgroup increased significantly, in contrast to the insignificant change in that of the LNA subgroup. Our results indicated the elevated role of LNA cells in the prokaryotic community and the enhanced viral lysis pressure on the total prokaryotes under oligotrophic conditions. This implies a weakened efficiency of carbon cycling within the microbial loop and enhanced viral lysis to shunt more carbon and energy flow in the future ocean, in which oligotrophication will be strengthened due to global warming.

## Introduction

Representing the largest living biomass reservoir (Suttle, 2007) and critical components of the microbial loop (Azam et al., 1983) in aquatic environments, prokaryotes play a crucial role in biogeochemical cycling in the global ocean. Consequently, even subtle changes in prokaryotic abundance and metabolic activity in response to varying environmental conditions would be amplified to substantially affect the structure and function of the marine ecosystem (Edwards and Richardson, 2004; Morán et al., 2010). Comprehensive knowledge of how the abundance and metabolic activity of prokaryotes and their regulating factors react to changes in the marine environment, such as the strengthened ocean oligotrophication due to the global warming-enhanced stratification (Agusti et al., 2017), is thus of critical importance.

The flow cytometry technique has revealed that prokaryotes cluster into high nucleic acid (HNA) cells and low nucleic acid (LNA) cells, and the two subgroups may be physiologically and ecologically distinct (Li et al., 1995; Gasol et al., 1999; Bouvier et al., 2007). Initially, the HNA cells were proposed to be the more dynamic and actively growing fraction of the prokaryotic community (Lebaron et al., 2001; Servais et al., 2003), while the LNA cells were considered to be the potentially dormant or dead cells (Jellett et al., 1996; Gasol et al., 1999). Such a view was later challenged when the LNA cells were found to exhibit substantial heterotrophic activity, comparable to that of the HNA cells (Zubkov et al., 2001; Longnecker et al., 2005; Scharek and Latasa, 2007; Huete-Stauffer and Morán, 2012). Indeed, the LNA cells, such as SAR11 bacteria, have small, streamlined genomes, and limited genetic repertoires and thereby a lower metabolic rate, weakened capability to utilize dissolved organic carbon (DOC), and narrower breadth of potential ecological niches (Giovannoni et al., 2005; Giovannoni, 2017). However, the compact genomes that LNA cells possess reduce their metabolic burden of replication under low resource availability, enabling them to gain a competitive advantage over HNA cells when or where nutrients are limited (Servais et al., 2003; Longnecker et al., 2005, 2006; Mojica et al., 2019). A natural hypothesis follows that the relative potential growth rate of LNA cells over HNA cells may increase as the aquatic environment becomes more oligotrophic. This hypothesis and the underlying roles of the potentially different regulating factors of the HNA and LNA subgroups, as of now, have not been investigated.

It is widely recognized that prokaryotic abundance and potential growth rate are dynamically controlled by both bottom-up (i.e., resource availability such as the inorganic nutrients) (Church, 2008) and top-down (i.e., mortality mediated by protozoan grazing and viral lysis) (Sanders et al., 1992; Thingstad and Lignell, 1997) factors. Among the top-down regulators, protozoa are the dominating grazers of prokaryotes and can transfer as much as 100% of the prokaryotic production to higher trophic levels in the ocean (Vazquez-Dominguez et al., 2006; Pearce et al., 2010), whereas viral lysis can contribute to 10–50% of daily prokaryotic mortality (Weinbauer et al., 2002) and is found to have a comparable contribution as that of protozoan grazing in particular situations (Fuhrman and Noble, 1995). In contrast to protozoan grazing, the viral lysis of prokaryotes causes the release of cellular material that is rich in organic matter into the surrounding environment for easy uptake by prokaryotes and thus redirects carbon and energy fluxes from higher trophic levels toward heterotrophic microbial processing (termed as “viral shunt”) (Suttle, 2005).

Previous studies demonstrated that the relative importance of protozoan grazing and viral lysis on prokaryotes varies by trophic status (Bettarel et al., 2004; Danovaro et al., 2008; Tsai et al., 2013b) and water depth (Nagata et al., 2010; Tsai et al., 2016; Lara et al., 2017). Furthermore, the relative contribution of the two top-down factors may be affected by the physiological state of different prokaryotic subgroups, which in turn shapes the community composition of prokaryotic assemblages (del Giorgio and Gasol, 2008). Several studies have observed that more active prokaryotic subgroups such as the HNA cells are preferentially grazed by protozoa (i.e., so-called selective grazing) (Gonzalez et al., 1990; del Giorgio et al., 1996; Sintes and del Giorgio, 2014; Baltar et al., 2016), whereas less active subgroups such as the LNA cells prevent heavy grazing because of their intrinsic low metabolic rates and small size, and thereby can achieve persistence and dominance under situations of strong grazing pressure (Segovia et al., 2018). Likewise, viruses are also recognized to exert disproportionately larger impacts on the dominant and fast-growing prokaryotic subgroup, following the “kill the winner” hypothesis, because viral replication largely relies on a host’s metabolic machinery and energy supply (Fuhrma and Suttle, 1993; Weinbauer, 2004; Chen et al., 2019a). However, strong top-down impact of viruses on SAR11 cells, the typical LNA prokaryotic subgroup, has been observed in an oligotrophic ocean (Zhao et al., 2013). This suggests that viruses can significantly impact LNA cells in contrast to protozoan grazing that is generally reported to place greater pressure on HNA over LNA cells. Such distinct regulating impacts by grazing and lysis on prokaryotic subgroups of different physiological states may contribute to the hypothesis mentioned above, namely, the elevated role of LNA cells in the prokaryotic community under more oligotrophic conditions. This, to the best of our knowledge, has not been explored yet.

This study aims to investigate how the abundance and potential growth rate of two distinct prokaryotic subgroups (i.e., HNA and LNA) and their regulating factors, including protozoan grazing and viral lysis, change under different trophic conditions in the natural environment. A series of modified dilution experiments were conducted along a transect spanning the nutrient-rich coastal zone to an area of nutrient-depleted open ocean in the northern South China Sea (SCS) in November 2016. Being the largest marginal sea in the northern Pacific, the SCS is an ideal testbed because it harbors both a river-dominated shelf region (i.e., the northern shelf is affected by large freshwater and terrestrial inputs from the Pearl River) and a vast area of an oligotrophic deep basin, facilitating the investigation of the differing regulatory impacts of protozoan grazing and viral lysis on the HNA and LNA subgroups under various nutrient conditions in the ocean.

## Materials and Methods

### Study Sites and Sampling

**FIGURE 1:**
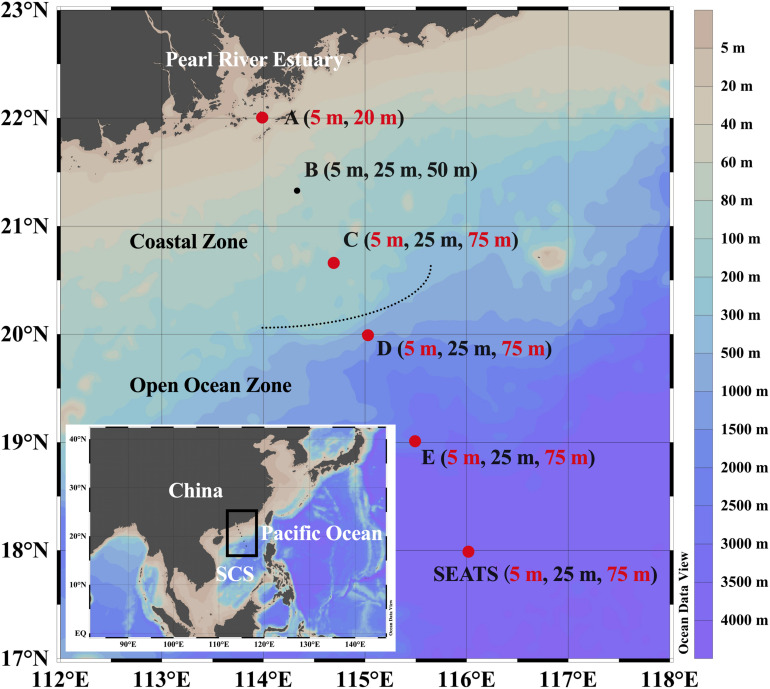
Geographic locations of the sampling stations in the northern South China Sea (SCS). Dots denote the sampling stations, and depths listed in the bracket denote the sampling depths of chemical variables and prokaryotic parameters of each station. Dots and texts in red indicate the stations and corresponding surface and subsurface depths where dilution experiments were conducted and heterotrophic nanoflagellate samples were collected. The map was generated using Ocean Data View software (Schlitzer, 2020).

Six sampling sites along a transect spanning the shelf water neighboring the Pearl River Estuary (PRE) to the deep basin of the northern SCS (Figure 1) were visited during a research cruise in November 2016. Water temperature and salinity were derived from a probe mounted on the CTD rosette (SBE9/11 plus, Sea-Bird Electronics Inc., United States) (Supplementary Table 1), and water samples were collected using 10-L Niskin bottles. The concentrations of nitrate (${\text{NO}}_{3}^{-}$), nitrite (${\text{NO}}_{2}^{-}$), silicate (${\text{SiO}}_{3}^{2-}$), and phosphate (${\text{PO}}_{4}^{3-}$) in the samples were determined by using an Auto Analysis III, AA3 instrument (Bran-Luebbe, Germany) in the main lab. Hereinafter, the sum of the nitrate and nitrite concentrations is referred to as the dissolved inorganic nitrogen (DIN). Samples for biological analysis, including prokaryotic and viral abundance, were prefiltered through 20-μm mesh filters to remove large particles and zooplankton.

### Abundance of Prokaryotes and Virus-Like Particles

Samples for determining abundance of prokaryotes and virus-like particles (VLP) were prepared via the following steps: 2-mL samples were fixed with glutaraldehyde at a final concentration of 0.5%, held at room temperature for 15 min in the dark, shock-frozen in liquid nitrogen, and stored at −80°C until analysis. Prokaryotic abundance was determined using flow cytometry (BD Accuri C6, United States) with a laser emitting light at 488 nm. Prior to analysis, the samples were thawed to room temperature, and 990-μL subsamples were stained in the dark with SYBR green I solution (Molecular Probes, United States) and then held at room temperature for 15 min (Gasol and del Giorgio, 2000). Prokaryotes were enumerated on flow cytometry by their signature in a plot of side scatter (SSC) versus green fluorescence (Supplementary Figure 1A). VLP abundance was determined using flow cytometry (Epics Altra II, Beckman Coulter, United States) after staining with SYBR green I (Molecular Probe, United States) based on an established protocol (Brussaard, 2004; Supplementary Figure 1B). Fluorescent beads with a diameter of 1 μm (Molecular Probes Inc., United States) were added as an internal standard to both the prokaryotic and VLP samples.

The high and low nucleic acid content (HNA and LNA) prokaryotic subgroups were discriminated based on their respective signature in the cytogram plot of SSC versus green fluorescence (Gasol et al., 1999). All flow cytometric data analysis was performed with the FlowJo vX.0.7 software (Tree Star, United States).

### Nanoflagellate Abundance

Heterotrophic nanoflagellate (HNF) abundance was measured by the following steps: 50-mL subsamples were fixed with glutaraldehyde at a final concentration of 1%, filtered through 0.45-μm polycarbonate black filters, and stained with DAPI (4,6-diamidino 2-phenylindole) at a final concentration of 10 μg mL^–1^ (Sherr et al., 1993; Yang et al., 2020). HNFs on the filters were counted along several transects using epifluorescence microscopy (Olympus BX51, Olympus America Inc., Center Valley, PA, United States) at ×1000 magnification. At least 50–100 HNF cells were counted in at least 25 fields per filter.

### Dilution Experimental Setup

A modified parallel dilution technique was utilized to estimate prokaryotic mortality mediated by protozoan grazing and viral lysis following Evans et al. (2003). Dilution experiments were carried out at the surface (5 m) and subsurface (20 m for the shallowest station A and 75 m for the remaining stations) layers of five stations (station A, C, D, E, and SEATS), as highlighted in Figure 1.

Briefly, seawater was first passed through a 20-μm mesh and then filtered through a tangential flow filtration system with a 0.2-μm and 30-kDa pore size polyvinylidene difluoride cartridges (Labscale, Millipore, United States) to generate the grazer-free and virus-free diluents, respectively (Supplementary Figure 2). The polycarbonate bottles used in the experiments were acid-cleaned with 10% HCl and rigorously rinsed with Milli-Q water. The diluents were added to 250-mL polycarbonate bottles in the correct proportions to generate a parallel t_0_ dilution series (20, 40, 60, and 100% of whole seawater). From each of these t_0_ bottles, triplicate 50-mL polycarbonate bottles were rinsed twice with diluents and then gently filled with diluents by siphoning to minimize physical damage to the grazers, viruses, and prokaryotes. Immediately after completing the above preparation steps, the 50-ml polycarbonate bottles (21 bottles in total for each experiment) were incubated in an on-deck incubator for 24 h with *in situ* light simulated by covering with a neutral density plastic sheet and under the same temperature as the seawater at the time they were sampled by running seawater at that temperature. Triplicate 2-mL samples were collected at the start and the end of incubation to measure the prokaryotic abundance.

### Interpretation of Dilution Experiments Results

The net growth rate of the prokaryotes (k, d^–1^) was calculated for each sample based on the prokaryotic abundance at the start and the end of the incubation experiment (N_t0_ and N_t_), assuming exponential growth (Landry and Hassett, 1982):

$$\text{k}=\ln\left(\frac{{\text{N}}_{\text{t}}}{{\text{N}}_{{\text{t}}_{0}}}\right)/\left(\text{t}-{\text{t}}_{0}\right)$$

The slope of the regression of the net growth rate versus the dilution factor for the grazer-free dilution series is interpreted as the protozoan grazing-mediated prokaryotic mortality (PMM), whereas the slope of the regression for the virus-free dilution series reflects the combined impact of protozoan grazing and viral lysis. Therefore, the viral lysis-mediated prokaryotic mortality (VMM) can be obtained from the difference between the slopes of the regression lines from the two-dilution series (Supplementary Figure 3). Potential prokaryotic growth (PPG) was determined as the y-intercept value of the regression line obtained from the virus-free dilution series. PPG, PMM, and VMM rates were calculated separately for the HNA and LNA subgroups.

### Statistical Analysis

GraphPad Prism 7 (GraphPad, United States) software was used to perform all statistical analyses. Briefly, a least-square regression analysis was conducted to analyze the relationship between the net growth rate and fractions of the grazer-free and virus-free dilution series. The PMM and VMM rates were analyzed using an *F*-test to assess whether a significant difference between the two sources of mortality existed, and a *P*-value less than 0.05 was considered significant. Shapiro–Wilk *W* tests were used to examine the data normality before analysis, and logarithm transformation was performed if necessary. Significant differences between samples were determined using paired *t*-tests. The relationships between the prokaryotic parameters and abiotic or biotic variables were examined using Spearman’s rank correlation analysis with a significance level (α) of 0.05.

## Results

### Physical and Chemical Characteristics

**FIGURE 2:**
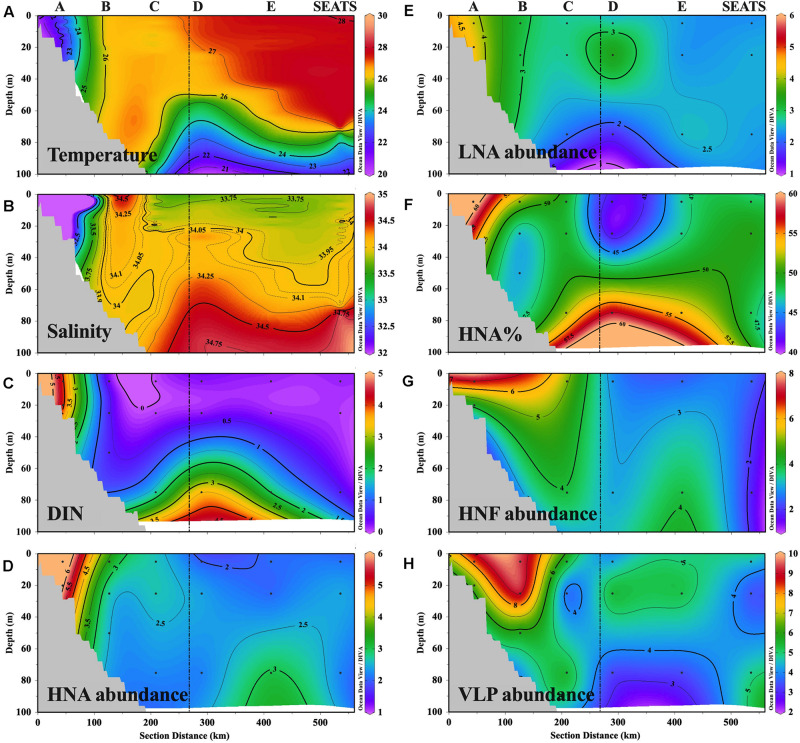
Transect distributions of **(A)** temperature (°C), **(B)** salinity, **(C)** DIN concentration (μmol L**^–^**^1^), **(D,E)** abundance (10^5^ cells ml**^–^**^1^) of HNA cells and LNA cells, **(F)** ratio of HNA cell abundance in total prokaryotic abundance (HNA%), **(G)** abundance (10^2^ cells ml**^–^**^1^) of heterotrophic nanoflagellate (HNF), and **(H)** abundance (10^6^ cells ml**^–^**^1^) of virus-like particles (VLP). The figure was plotted with Ocean Data View software (Schlitzer, 2020).

The temperature of the upper 70 m water generally increased from nearshore station A to offshore station SEATS (Figure 2A and Supplementary Table 1). Vertically, the temperatures at shallower stations A and B were almost homogeneous throughout the entire water column, while the other offshore stations exhibited a mixed layer of approximately 60∼70 m thick lying above the colder deep waters (Figure 2A). The salinity of the upper 70 m water showed a pronounced gradient that was impacted by the fresh river plume, with station A affected by the plume possessing the lowest salinity and the remaining stations with minor plume influence having a higher salinity (Figure 2B). Similar to temperature, the vertical distribution of the salinity at stations A and B was more homogeneous than that of the other offshore stations, where the salinity generally increased with depth (Figure 2B).

The spatial distribution of DIN along the transect largely mirrored that of the physical variables, with higher and vertically more mixed concentrations at the station affected by the nutrient-rich river plume (i.e., A has the highest surface DIN, with mean ± standard deviation of 4.15 ± 0.06 μmol L^–1^), whereas distinctly lower surface (0.11–0.35 μmol L^–1^) but higher subsurface concentrations (0.55–3.96 μmol L^–1^) were observed at the other offshore stations that were minimally affected by the plume (Figure 2C). The distribution pattern of silicate was similar to that of DIN while phosphate concentrations did not show a clear spatial distribution pattern (Supplementary Figure 4).

### Microbial Abundance

Similar to the physical and chemical characteristics of the sample locations, the spatial distribution of HNA abundance also showed an environmental gradient along the transect that was shaped by river inputs. Namely, HNA abundance was distinctly higher at station A and lower at the offshore stations, except for a relatively high subsurface value at 75 m depth at E (Figure 2D). The environmental gradient of the distribution of LNA abundance was weak, with the peak at station A (Figure 2E). The percentage of HNA abundance in total prokaryotic abundance (HNA%) was higher at A (60 ± 2%) and the subsurface layer of the offshore stations C (52%), D (58%), and E (54%) where the DIN concentration was higher (Figure 2F). The distribution pattern of the HNF (Figure 2G) and VLP abundance (Figure 2H) largely mirrored that of HNA and LNA abundance, with the values generally decreasing offshore from A toward SEATS (Supplementary Table 1).

### Potential Prokaryotic Growth Rate

**FIGURE 3:**
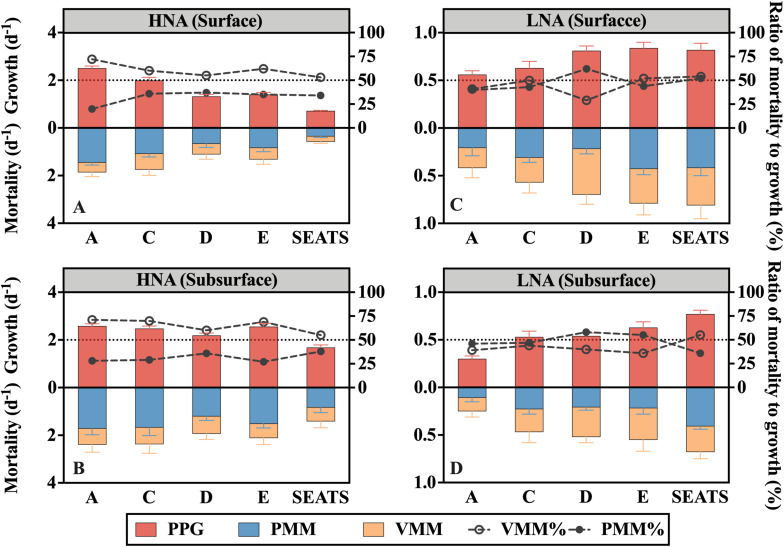
Potential prokaryotic growth rate (PPG), mortality rate mediated by protozoan grazing (PMM) and viral lysis (VMM), and ratios of PMM (PMM%, filled dots) or VMM (VMM%, empty dots) to PPG for the **(A,B)** HNA subgroup and **(C,D)** LNA subgroup at stations along the transect at the surface and subsurface layers. The horizontal dashed line denotes the ratio of 50%.

The potential growth rate of the HNA group (PPG-H) at the surface generally decreased offshore (Figure 3A), while no apparent along-transect trend was found for subsurface PPG-H (Figure 3B). The subsurface average (2.30 ± 0.38 d^–1^) was significantly higher than the surface average (1.59 ± 0.68 d^–1^) (paired *t*-test, *P* < 0.05), where the maximum subsurface PPG-H (at A) was nearly fourfold greater than the minimum surface PPG-H (at SEATS).

The potential growth rate of the LNA subgroup (PPG-L) at the surface generally increased offshore from A to E but slightly declined to SEATS (Figure 3C), while PPG-L in the subsurface water persistently increased from A to SEATS (Figure 3D). The surface average of PPG-L (0.72 ± 0.12 d^–1^) was significantly (paired *t*-test, *P* < 0.05) higher than the subsurface average (0.55 ± 0.17 d^–1^).

**FIGURE 4:**
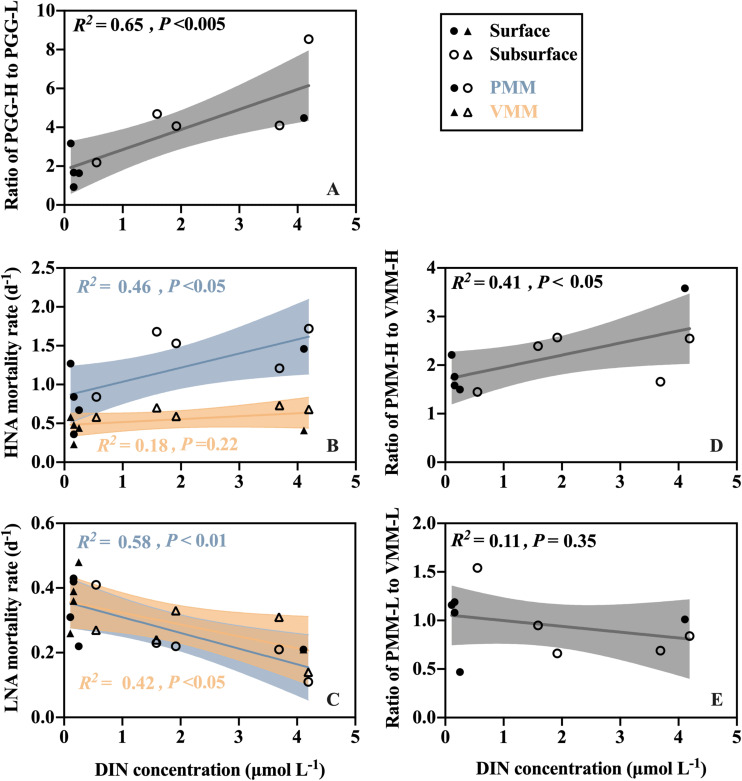
Linear correlations between DIN concentration and **(A)** the ratio of HNA prokaryotic potential growth rate (PPG-H) to LNA prokaryotic potential growth rate (PPG-L), **(B,C)** the protozoan grazing-mediated mortality rate (PMM) and viral lysis-mediated mortality rate (VMM) for the **(B)** HNA and **(C)** LNA subgroups, and **(D,E)** the ratio of protozoan grazing-mediated mortality (PMM) to viral lysis-mediated mortality (VMM) for the **(D)** HNA and **(E)** LNA subgroups.

The ratio of potential growth rates of HNA to LNA (PPG-H/PPG-L) reflects the relative activity of the HNA cells to the LNA cells. The maximum ratio was recorded at the subsurface layer of A (8.54), and the minimum was at the surface layer of SEATS (0.87) (Supplementary Table 2). Linear regression analysis of all surface and subsurface measurements further showed that the ratio of PPG-H/PPG-L was positively related to the DIN concentration (*R*^2^ = 0.65, *P* < 0.005) (Figure 4A).

### Prokaryotic Grazing-Mediated and Lysis-Mediated Mortality Rate

Protozoan grazing-mediated mortality of the HNA subgroup (PMM-H) was higher at nearshore station A than at the offshore stations and was significantly higher in the subsurface than surface layers (paired *t*-test, *P* < 0.05) (Figure 3A). No clear spatial trend was found for viral lysis-mediated mortality of the HNA subgroup (VMM-H) except that the subsurface values were generally higher than the surface values (Figure 3B). Linear regression analysis of all surface and subsurface measurements further showed that PMM-H was positively related to the DIN concentration (*R*^2^ = 0.46, *P* < 0.05) while there is no significant correlation between VMM-H and DIN (Figure 4B). The sum of PMM-H and VMM-H accounted for approximately 87∼96% and 93∼99% of the HNA prokaryotic production in the surface and subsurface layers, respectively. Specifically, protozoan grazing consumed more than 50% of the HNA prokaryotic production in both the surface and subsurface layers of all stations (Figures 3A,B).

**FIGURE 5:**
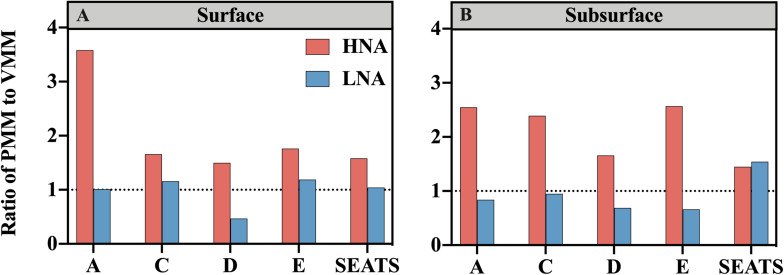
Ratios of protozoan grazing-mediated mortality (PMM) to viral lysis-mediated mortality (VMM) for the HNA subgroup (red bars) and LNA subgroup (blue bars) at stations along the transect at the **(A)** surface and **(B)** subsurface layers. The horizontal dashed line denotes the ratio of 1.

The ratio of PMM-H to VMM-H (PMM-H/VMM-H) represents the relative contribution of protozoan grazing over viral lysis to the mortality of the HNA subgroup, where a ratio value higher than 1 indicates greater contribution by grazing than lysis and vice versa. The ratio of PMM-H/VMM-H was distinctly higher than 1 for all surface and subsurface samples (Figure 5). At the surface, the ratio was remarkably higher at nearshore station A (3.58) than at the offshore stations (average was 1.63 ± 0.11) (Figure 5A), while no clear spatial trend was found for the ratio values at subsurface (range from 1.45 to 2.57) (Figure 5B). After clumping all surface and subsurface measurements, linear regression analysis showed that the PMM-H/VMM-H ratio was positively related to the DIN concentration (*R*^2^ = 0.41, *P* < 0.05) (Figure 4D).

The protozoan grazing-mediated mortality of the LNA subgroup (PMM-L) did not show a clear trend along the transect, although a significant difference was found between layers (paired *t*-test, *P* < 0.05), with the surface value of each station generally higher than its subsurface counterpart (Figures 3C,D). The distribution pattern of viral lysis-mediated mortality of the LNA group (VMM-L) in the surface waters was similar to VMM-H, increasing offshore (Figure 3C), whereas no clear spatial trend was found for the subsurface VMM-L (Figure 3D). Based on the linear regression analysis of all surface and subsurface measurements, both PMM-L and VMM-L were negatively related to the DIN concentration (*R*^2^ = 0.58, *P* < 0.01 and *R*^2^ = 0.42, *P* < 0.05) (Figure 4C). The sum of PMM-L and VMM-L accounted for approximately 81∼105% and 85∼97% of the LNA prokaryotic production at surface and subsurface layers, respectively. Specifically, protozoan grazing consumed less than 50% of the LNA prokaryotic production at the majority of the stations (Figures 3C,D).

No clear spatial trend was found for the ratio of protozoan grazing to viral lysis of the LNA subgroup (PMM-L/VMM-L) in the surface (Figure 5A) or subsurface waters (Figure 5B). The PMM-L/VMM-L averaging over the entire transect (0.96 ± 0.31) was very close to 1, which was much lower than the transect average of PMM-H/VMM-H (2.07 ± 0.06). In contrast to the HNA subgroup, no significant linear correlation was found between PMM-L/VMM-L ratio and DIN concentration (Figure 4E).

### Abiotic and Biotic Drivers of Prokaryote Abundance and Potential Growth Rate

**FIGURE 6:**
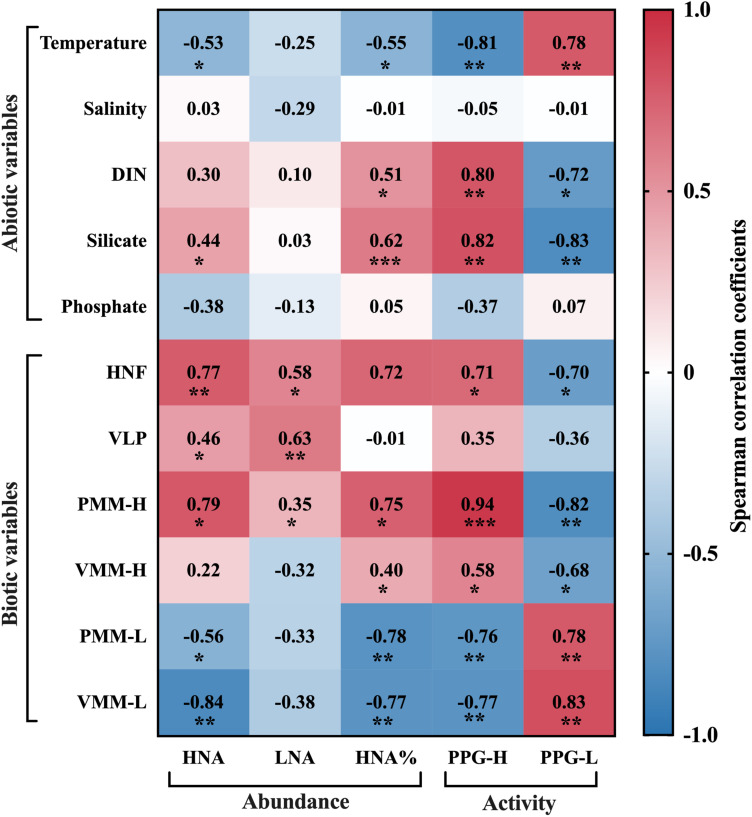
Spearman’s rank correlation coefficients between prokaryotic parameters and abiotic and biotic variables, where * denotes that the correlation is significant at the *P* < 0.05 level, ** at the *P* < 0.01 level, and *** at the *P* < 0.001 level.

Spearman’s rank correlation analysis was used to reveal the potential drivers of prokaryote abundance and potential growth rate (Figure 6). HNA abundance was strongly, positively related to silicate concentration (*r* = 0.44, *P* < 0.05), HNF abundance (*r* = 0.77, *P* < 0.01), VLP abundance (*r* = 0.46, *P* < 0.05), and protozoan grazing on the HNA subgroup (*r* = 0.73, *P* < 0.05) and was negatively related to temperature (*r* = −0.53, *P* < 0.05). Conversely, LNA abundance was not related to any nutrient but was strongly, positively related to HNF abundance (*r* = 0.58, *P* < 0.05) and VLP abundance (*r* = 0.63, *P* < 0.01). HNA% exhibited similar correlation analysis results as the HNA abundance except that HNA% was also significantly positively related to DIN (*r* = 0.51, *P* < 0.05) but not VLP abundance, despite the positive correlation between the abundance of VLP and each subgroup.

The potential growth rate of the HNA group was positively related to DIN and silicate concentrations (*r* = 0.80 and 0.82, *P* < 0.01), HNF abundance (*r* = 0.71, *P* < 0.05), and protozoan grazing and viral lysis of the HNA subgroup (*r* = 0.94, *P* < 0.001 and *r* = 0.58, *P* < 0.05). Specifically, the correlation between PPG-H and grazing was more significant than that between PPG-H and lysis. In contrast to PPG-H, PPG-L showed negative correlations with DIN and silicate concentrations (*r* = −0.72, *P* < 0.05 and *r* = −0.83, *P* < 0.01) and had comparable significant, positive correlations with protozoan grazing and viral lysis on the LNA subgroup (*r* = 0.78 and *r* = 0.83, *P* < 0.01).

## Discussion

The cross-shore transect in the northern SCS possessed a clear nutrient gradient, which provides an ideal testbed to investigate how the abundance and potential growth rate of HNA and LNA prokaryotes and their regulating factors (i.e., protozoan grazing and viral lysis) respond to various nutrient conditions. The transect survey was carried out in the early winter season when the relatively strong northeast wind prevailed, and the Pearl River discharge was less than the annual mean, resulting in a narrower offshore plume extension (Yin et al., 2001). This fact was reflected by the pronounced environmental gradient shown in the upper water along the surveyed transect, which transited from the colder, fresher, and nutrient-rich water at nearshore station A to the warmer, saltier, and nutrient-poor water at offshore SEATS. Vertically, while the shallower stations A and B (<75 m) were well mixed, the other deeper stations offshore exhibited a distinct vertical gradient with nutrient-depleted surface water lying on the nutrient-replete subsurface water (>70 m).

### Differential Responses of HNA and LNA Prokaryotes to Varying Trophic Status

The abundance of the HNA and LNA subgroups responded differently to the abiotic and biotic variables. Specifically, the spatial distribution of HNA abundance exhibited an environmental gradient along the transect that was similar to the physical and chemical characteristics, i.e., decreasing from coastal to open ocean, and was generally higher at the subsurface than the surface waters of the open ocean. In contrast, the spatial distribution of LNA abundance showed a weaker gradient along the transect. The different responses of the abundance of the two subgroups to environmental factors were further reflected by the fact that HNA abundance was significantly correlated with both silicate concentration and temperature, while LNA abundance was not related to any abiotic factor. It follows that the HNA subgroup is more responsive to environmental variations than the LNA subgroup. A similar phenomenon was observed in the Atlantic Ocean where HNA abundance changed more than twofold between seasons, acting similar to an r-strategist or opportunist, while the LNA subgroup demonstrated more equilibrium dynamics over the same period, likely following the k-strategy (Mojica et al., 2019). This tendency can be attributed to the higher genome complexity of HNA than LNA cells, which enables the HNA subgroup to have a higher capacity for exploiting pulses of nutrients (Azam, 1998) as well as the ability to occupy a greater variety of ecological niches (Philippot et al., 2010; Schattenhofer et al., 2011). Indeed, the HNA subgroup consists of more versatile and faster-growing cells and possesses larger genomes, while the LNA subgroup is commonly dominated by oligotrophs, which have smaller, less flexible genomes (Zubkov et al., 2001; Longnecker et al., 2005; Mary et al., 2006; Schattenhofer et al., 2011). The compact genomes that LNA cells possess place them at a competitive disadvantage against HNA cells in nutrient-rich conditions, but their reduced metabolic burden of replication gains them a competitive advantage over HNA cells in an oligotrophic environment or during periods of limited resource availability (Mojica et al., 2019). This perspective is well supported by our data, which showed that the relative contribution of HNA to total prokaryotic abundance, HNA%, significantly decreased with decreasing DIN and silicate concentration.

Similar to HNA abundance, the spatial distribution of HNA prokaryotic potential growth rate (PPG-H) showed a clear environmental gradient, largely mirroring the distribution pattern of DIN and silicate concentration, i.e., declining offshore, and was significantly higher at the nutrient-rich subsurface of the open ocean than the nutrient-poor surface of the open ocean. The spatial distribution of LNA prokaryotic potential growth rate (PPG-L) showed the opposite pattern to PPG-H and nutrient distributions, which generally increased from coastal to open ocean and was higher at the surface than subsurface waters of the open ocean. This reverse response of HNA and LNA cells to nutrient levels is also reflected by the significant positive correlations between PPG-H and DIN and silicate concentration in contrast to the significant negative correlations between the PPG-L and DIN and silicate concentration. As a result, a significant positive linear relation was found between the PPG-H/PPG-L ratio and DIN concentration, suggesting that the relative activity of the HNA cells decreased while that of the LNA cells increased under oligotrophication. This finding is consistent with other studies that found that open ocean oligotrophic communities, compared to coastal or shelf communities, have a higher proportion of heterotrophic activity ascribed to the LNA subgroup (Servais et al., 2003; Longnecker et al., 2005, 2006). The contrasting response of HNA and LNA potential growth rate across nutrient gradients is also attributable to the higher genome complexity of the HNA than LNA cells, which allows HNA to gain a competitive advantage in situations of abundant resources while placing LNA cells at an advantageous state in the resource-limited environment, as discussed above (Zubkov et al., 2001; Schattenhofer et al., 2011).

The increasing fraction of LNA abundance in total prokaryotic abundance (indicated by declining HNA%) and increasing relative activity of LNA cells (indicated by declining PPG-H/PPG-L ratio) with decreasing nutrients revealed in our data may suggest a reduced capacity of the total prokaryotic community to degrade and utilize DOC under the more oligotrophic conditions. This is associated with that LNA cells, compared to the HNA cells, have lower metabolic rate and weaker capability to degrade and utilize DOC due to their small streamlined genomes, limited genetic repertoires, and simpler extracellular hydrolase system (Giovannoni et al., 2005; Giovannoni, 2017). A similar point of view has been raised by Zubkov et al. (2004) who hypothesized that LNA cells only consume the labile fraction of organic nutrients, while HNA cells also feed on more refractory sources.

### Elevated Contribution of Viral Lysis With Declining Nutrient

Protozoan grazing and viral lysis are well recognized as the major agents of the top-down control of prokaryotes (Thingstad and Lignell, 1997). Previous studies across different aquatic environments (Calbet et al., 2001; Weinbauer, 2004; Corte et al., 2012; Tsai et al., 2013a; Baltar et al., 2016; Wigington et al., 2016; Chen et al., 2019b; Li et al., 2019; Wei et al., 2019) have indicated significant positive correlations between prokaryote abundance with HNF and/or VLP abundance. Our data further revealed that the close correlation with HNF and VLP abundance also existed in each prokaryotic subgroup, the HNA and LNA. The tight coupling between prokaryotic subgroups and their respective top-down control factors is additionally supported by the high percentages of protozoan grazing and viral lysis in accounting for HNA prokaryotic production (87–99%) and LNA prokaryotic production (81–105%).

Importantly, our results indicated the contrasting regulating impacts of protozoan grazing and viral lysis on the abundance and potential growth rates of the HNA and LNA subgroups. The protozoan grazing profoundly impacted HNA abundance but not the LNA abundance, as suggested by the correlation analysis. On the contrary, VLP abundance was found to be significantly, positively correlated with both HNA and LNA abundance and the correlation between VLP abundance and LNA subgroup was stronger and more significant than that of HNA. The contrasting regulatory role of protozoan grazing and viral lysis on the HNA and LNA subgroups is also reflected in the prokaryotic potential growth rates. The HNA subgroup exhibited a stronger and more significant correlation with protozoan grazing than viral lysis, whereas the correlations between the LNA subgroup and grazing and lysis were more or less equivalent. In other words, the HNA cells were subjected to greater top-down control from grazing than lysis, while the LNA cells experienced equivalent pressures from the two regulators. This is well evidenced by the higher grazing to virus lysis ratio for HNA (ranging from 1.45 to 3.58) than for the LNA subgroup (0.47 to 1.54). We attribute the greater protozoan grazing pressure on the HNA than LNA cells to the selective grazing strategy of protozoa. Namely, the protozoa preferentially graze HNA cells that belong to the actively growing portion of the prokaryotic assemblage (del Giorgio et al., 1996; Baltar et al., 2016), whereas the less active prokaryote subgroup, such as the LNA cells, are less heavily grazed due to their intrinsically low metabolic rates and small size (Segovia et al., 2018). On the contrary, viral lysis may have weaker preference to different prokaryotic subgroups than grazing does, as evidenced by the lower VMM-H/VMM-L ratio than PMM-H/PMM-L ratio (Supplementary Table 2). Previously, high abundance of viruses to infect SAR11 population, the typical LNA prokaryotic subgroup, has been observed widespread in the oligotrophic ocean (Zhao et al., 2013), suggesting that viral lysis can profoundly impact LNA prokaryotic subgroup. That discovery has stimulated a hot debate on whether the abundant viruses infecting SAR11 cells is contradictory with the “Kill the Winner” theory (Giovannoni et al., 2013; Våge et al., 2013). For example, Våge et al. (2013) suggested that the high abundance of SAR11 cells and their phage could co-exist when strains of all species are dispersed over the entire growth-rate axis; under such scenario, most of the viruses would lysis the fastest growing strains (i.e., the “winner strains”) to keep them at low abundance while allowing the slow-growing strains to dominate consequently, which is consistent with the “Kill the Winner” theory. More future works are still needed to unveil the mechanism of viruses infecting the less active but abundant prokaryotic subgroup. Nevertheless, the more comparable regulatory impact of grazing and lysis on LNA cells as revealed in our data is due to a combination of the weaker preference of viral lysis on HNA over LNA cells and the relatively lower protozoan grazing pressure on LNA cells.

Another interesting finding is that the relative role of protozoan grazing to viral lysis (indicated by the ratio of grazing to lysis) in regard to the HNA and LNA subgroups responded differently to varying nutrient levels. Specifically, the relative importance of grazing on the HNA subgroup declined with decreasing nutrient concentration, which may be explained by the reduced food quality or availability under lower nutrients, because food quality (e.g., cell size, cell surface properties) and availability are known to influence protozoan grazers and grazing rates (Monger and Landry, 1991; Christaki et al., 1998; Monger et al., 1999). The declining food availability is supported by our data that abundance of picoeukaryote, one of the primary producers supplying fresh DOC, significantly decreased with decreasing DIN concentration (*R*^2^ = 0.32, *P* < 0.05; Supplementary Figure 5). On the contrary, both grazing and lysis on the LNA subgroup significantly increased with declining nutrient concentration because the LNA subgroup gains a more competitive advantage as the nutrients become limited in the environment. This identical direction of change in grazing and lysis on LNA cells with varying nutrient levels largely explains why there was no significant change in the ratio of grazing to lysis on the LNA subgroup along the varying trophic conditions. Nevertheless, given the relatively lower impact ratio of grazing to lysis on LNA compared to the HNA subgroup, an increase in the LNA contribution to the prokaryotic community together with an increase in impact ratio of grazing to lysis on HNA subgroup with decreasing nutrient levels, as our data revealed, imply that the relative impact of grazing on the mortality of the total prokaryotic community will decrease while that of viral lysis will increase under more oligotrophic conditions.

### Implications for Carbon Cycling and Sequestration in Future Ocean

**FIGURE 7:**
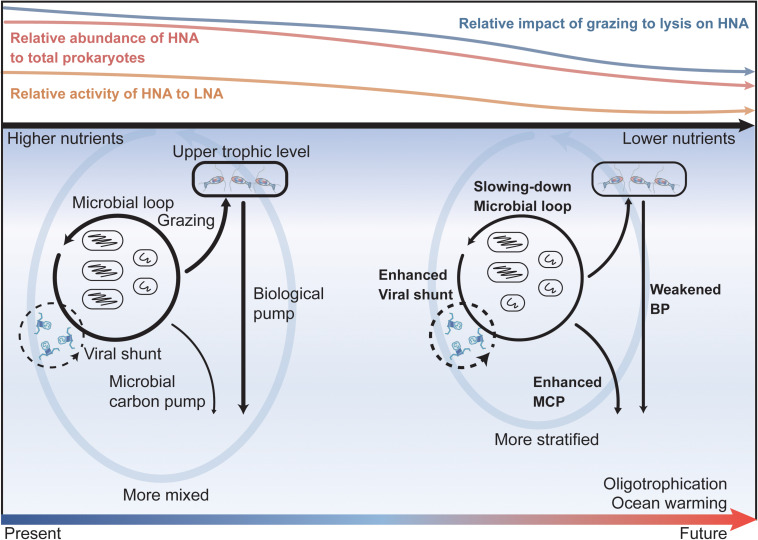
A summary schematic and implications for future ocean oligotrophication scenarios. The colored curves on the top sketch the responses of prokaryotic abundance, activity (indicated by prokaryotic potential growth rate), and regulating top-down impacts to the declining nutrient levels revealed in our study. Namely, as the nutrient level decreases, the relative contribution of HNA abundance to the total prokaryotes (red curve), the relative activity of the HNA cells to LNA cells (orange curve), and the relative impact of grazing to lysis on the HNA cells (blue curve) all decrease. The lower column of the figure depicts the potential changes (denoted by the width of the black arrows), implied from our study, in the efficiency of the microbial loop, viral shunt, biological pump (BP), and microbial carbon pump (MCP) under future ocean warming and oligotrophication. Driven by global warming, the future ocean will be warmer, more stratified, and have a weaker vertical nutrient supply and an enhanced oligotrophication in the upper ocean. Under the strengthened ocean oligotrophication state, the efficiency of the microbial loop will be retarded, and viral shunt will be enhanced while the carbon and energy directed to the upper trophic level will be reduced, which will consequently enhance the efficiency of the microbial carbon pump while weakening the efficiency of the biological pump in carbon sequestration.

Our data revealed that as the nutrient concentrations declined, the proportion of LNA abundance to total prokaryotes, the relative prokaryotic potential growth rate of the LNA to HNA cells, and the relative impact of viral lysis to protozoan grazing on total prokaryotes all increased. Such responses of prokaryotic abundance and potential growth rate and their top-down regulating factors to the decreasing nutrient levels have important implications for carbon cycling and sequestration in the future ocean (Figure 7). The anticipated ocean warming under global climate change is expected to enhance oligotrophication of the upper ocean as a consequence of the reduced diffusive vertical nutrient supply driven by the strengthening stratification (Hoegh-Guldberg and Bruno, 2010; Agusti et al., 2017). Under this increased oligotrophication, the ocean is expected to embrace a future of retarding carbon cycling in the microbial loop (due to the elevated role of the LNA subgroup in regard to the total prokaryotic community) and enhanced viral shunt to retain the carbon and energy flow within the microbial loop (due to the higher relative contribution of viral lysis to the mortality of the total prokaryotes). While these changes will weaken the efficiency of the biological pump following the reduced energy and carbon fluxes directing to the upper trophic levels, they will also promote the accumulation of refractory DOC, according to the theory of the microbial carbon pump (MCP) (Jiao et al., 2010, 2014, 2018), and thus enhance carbon sequestration in the ocean. This, in turn, will stimulate negative feedback to the global warming associated with the accelerated release of anthropogenic CO_2_.

## Conclusion

This study revealed that the potential growth rate of the HNA and LNA subgroups responded reversely along the nutrient gradient. Namely, as the nutrient concentrations decreased, the HNA potential growth rate significantly decreased, while that of the LNA markedly increased, thus yielding an increasing relative potential growth rate of the LNA subgroup with declining nutrient levels. Furthermore, there existed contrasting regulatory impacts of protozoan grazing and viral lysis on the HNA and LNA subgroups, where the HNA subgroup experienced higher pressure from grazing than lysis while the LNA subgroup was subjected to an equivalent impact from grazing and lysis. The relative role of lysis to grazing on the HNA subgroup increased with decreasing nutrients. Taken together, our results revealed an elevated contribution of LNA cells to the prokaryotic community and a relatively greater virus-mediated mortality pressure on total prokaryotes under more oligotrophic conditions. This implies a weakened efficiency of carbon cycling within the microbial loop and enhanced viral lysis to shunt more carbon and energy flow in the anticipated future ocean oligotrophication driven by global warming.

## Data Availability Statement

The raw data supporting the conclusions of this article will be made available by the authors, without undue reservation.

## Author Contributions

CH designed and performed the experiments under the supervision of DX and NJ. CH carried out the formal analysis with assistance and inputs from XC and LY. CH wrote the manuscript with contributions from all co-authors. All authors contributed to the article and approved the submitted version.

## Conflict of Interest

The authors declare that the research was conducted in the absence of any commercial or financial relationships that could be construed as a potential conflict of interest.
