# Supplementary material for: Elevated Contribution of Low Nucleic Acid Prokaryotes and Viral Lysis to the Prokaryotic Community Along the Nutrient Gradient From an Estuary to Open Ocean Transect
Source: Front Microbiol. 2020 Dec 15;11:612053. doi: 10.3389/fmicb.2020.612053 (PMC7793805; doi:10.3389/fmicb.2020.612053)
Supplement: Supplementary file 1 [file Data_Sheet_1.docx]

**Supplementary material**

**Table S1.** Location and environmental parameters of investigated stations along the transect in the northern South China Sea.

| **Stations** | **Longitude** | **Latitude** | **Depth** | **Temperature** | **Salinity** | **DIN** | **SiO_3_^2-^** | **PO_4_^3-^** | **HNA** | **LNA** | **Prokaryote** | **HNA%** | **HNF** | **PFR** | **VLP** | **VPR** |
| --- | --- | --- | --- | --- | --- | --- | --- | --- | --- | --- | --- | --- | --- | --- | --- | --- |
|  | **°E** | **°N** | **m** | **℃** |  | $\boldsymbol{\mu mol}\mathbf{L}^{\mathbf{-1}}$ | $\boldsymbol{\mu mol}\mathbf{L}^{\mathbf{-1}}$ | $\boldsymbol{\mu mol}\mathbf{L}^{\mathbf{-1}}$ | **10^5** $\mathbf{cell m}\mathbf{L}^{\mathbf{-1}}$ | **10^5** $\mathbf{cell m}\mathbf{L}^{\mathbf{-1}}$ | **10^5** $\mathbf{cell m}\mathbf{L}^{\mathbf{-1}}$ |  | **10^2** $\mathbf{cell m}\mathbf{L}^{\mathbf{-1}}$ |  | **10^6** $\mathbf{cell m}\mathbf{L}^{\mathbf{-1}}$ |  |
| A | 114.00 | 22.00 | 5 | 22.98 | 30.40 | 4.11 | 4.47 | 0.24 | 6.71 | 4.17 | 10.88 | 0.62 | 7.15 | 15.21 | 8.30 | 7.63 |
|  |  |  | 20 | 22.30 | 31.24 | 4.19 | 4.79 | 0.27 | 6.13 | 4.35 | 10.47 | 0.59 | 4.03 | 25.95 | 6.40 | 6.11 |
| B | 114.33 | 21.33 | 5 | 26.28 | 34.08 | 0.20 | 1.63 | 0.01 | 3.42 | 3.16 | 6.58 | 0.52 |  |  | 9.25 | 14.05 |
|  |  |  | 25 | 26.16 | 34.07 | 0.50 | 2.02 | 0.04 | 2.65 | 3.05 | 5.70 | 0.46 |  |  | 9.55 | 16.75 |
|  |  |  | 50 | 26.16 | 34.09 | 0.57 | 2.35 | 0.04 | 2.53 | 3.02 | 5.55 | 0.46 |  |  | 5.52 | 9.95 |
|  | 114.67 | 20.66 | 5 | 26.58 | 33.74 | 0.11 | 1.74 |  | 2.63 | 2.77 | 5.40 | 0.49 | 5.10 | 10.59 | 5.60 | 10.37 |
|  |  |  | 25 | 26.71 | 34.00 | 0.12 | 1.89 |  | 2.68 | 2.77 | 5.45 | 0.49 |  |  | 3.54 | 6.50 |
|  |  |  | 75 | 26.26 | 34.05 | 1.59 | 3.13 | 0.11 | 2.26 | 2.11 | 4.37 | 0.52 | 4.01 | 10.90 | 5.58 | 12.78 |
| D | 115.00 | 20.00 | 5 | 27.27 | 33.62 | 0.25 | 1.54 | 0.29 | 1.98 | 2.68 | 4.66 | 0.42 | 2.81 | 16.59 | 4.22 | 9.05 |
|  |  |  | 25 | 27.03 | 33.90 | 0.12 | 1.73 | 0.53 | 2.45 | 3.50 | 5.95 | 0.41 |  |  | 5.56 | 9.33 |
|  |  |  | 75 | 22.07 | 34.71 | 3.69 | 3.77 | 0.70 | 2.19 | 1.60 | 3.79 | 0.58 | 2.98 | 12.70 | 3.23 | 8.53 |
| E | 115.50 | 19.00 | 5 | 27.77 | 33.61 | 0.16 | 1.57 | 0.16 | 2.09 | 2.40 | 4.48 | 0.47 | 2.43 | 18.45 | 4.94 | 11.02 |
|  |  |  | 25 | 27.65 | 33.72 | 0.14 | 1.85 | 0.20 | 2.01 | 2.31 | 4.31 | 0.47 |  |  | 5.33 | 12.35 |
|  |  |  | 75 | 23.32 | 34.59 | 1.92 | 3.00 | 0.15 | 3.08 | 2.58 | 5.66 | 0.54 | 3.99 | 14.20 | 3.35 | 5.92 |
| SEATS | 116.00 | 18.00 | 5 | 27.96 | 33.46 | 0.16 | 1.91 | 0.34 | 2.11 | 2.32 | 4.42 | 0.48 | 2.42 | 18.31 | 4.60 | 10.41 |
|  |  |  | 25 | 28.00 | 33.93 | 0.10 | 1.61 | 0.06 | 2.46 | 2.49 | 4.95 | 0.50 |  |  | 3.40 | 6.87 |
|  |  |  | 75 | 23.77 | 34.81 | 0.55 | 2.97 | 0.22 | 2.22 | 2.40 | 4.61 | 0.48 | 1.75 | 26.37 | 4.87 | 10.55 |

*HNA, high nucleic acid cell; LNA, low nucleic acid cell; HNA%, The percentage of HNA abundance in total prokaryotic abundance; HNF, heterotrophic nanoflagellate; PFR, prokaryote to HNF ratio; VLP, virus-like particle; VPR, VLP to prokaryote ratio.*

**Table S2.** Dilution experiment-based potential prokaryotic growth rates (PPG), protozoan grazing (PMM)- and viral lysis (VMM)-mediated mortality for the high nucleic acid (HNA) and low nucleic acid (LNA) subgroups, ratio of PMM to VMM, ratio of HNA prokaryotic activity (PPG-H) to LNA prokaryotic activity (PPG-L), ratio of protozoan grazing mediated HNA mortality (PMM-H) to LNA mortality (PMM-L) and ratio of viral lysis mediated HNA mortality (VMM-H) to LNA mortality (VMM-L). The standard error is given in the parentheses.

| **Stations** | **Depth** | **HNA** | | | |  | **LNA** | | | | **PPG-H**  **/PPG-L** | **PMM-H**  **/PMM-L** | **VMM-H**  **/VMM-L** |
| --- | --- | --- | --- | --- | --- | --- | --- | --- | --- | --- | --- | --- | --- |
|  | **（m）** | **PPG (d^-1^)** | **PMM (d^-1^)** | **VMM (d^-1^)** | **PMM**  **/VMM** |  | **PPG (d^-1^)** | **PMM (d^-1^)** | **VMM (d^-1^)** | **PMM**  **/VMM** |  |  |  |
| A | 5 | 2.51 (0.09) | 1.46 (0.10) | 0.41 (0.18) | 3.58 |  | 0.56 (0.04) | 0.21 (0.08) | 0.21 (0.10) | 1.01 | 4.48 | 6.81 | 1.93 |
|  | 20 | 2.58 (0.09) | 1.72 (0.25) | 0.68 (0.31) | 2.55 |  | 0.30 (0.03) | 0.11 (0.04) | 0.14 (0.06) | 0.84 | 8.54 | 15.02 | 4.97 |
| C | 5 | 1.99 (0.13) | 1.09 (0.13) | 0.66 (0.24) | 1.66 |  | 0.63 (0.07) | 0.31 (0.05) | 0.26 (0.11) | 1.16 | 3.18 | 4.16 | 2.20 |
|  | 75 | 2.48 (0.11) | 1.68 (0.34) | 0.70 (0.38) | 2.39 |  | 0.53 (0.06) | 0.23 (0.05) | 0.24 (0.11) | 0.95 | 4.69 | 7.33 | 2.93 |
| D | 5 | 1.33 (0.08) | 0.67 (0.15) | 0.44 (0.20) | 1.50 |  | 0.81 (0.05) | 0.22 (0.05) | 0.48 (0.10) | 0.47 | 1.64 | 2.97 | 0.92 |
|  | 75 | 2.19 (0.09) | 1.21 (0.17) | 0.73 (0.24) | 1.66 |  | 0.54 (0.03) | 0.21 (0.03) | 0.31 (0.06) | 0.69 | 4.10 | 5.74 | 2.38 |
| E | 5 | 1.41 (0.09) | 0.84 (0.15) | 0.48 (0.20) | 1.76 |  | 0.84 (0.06) | 0.43 (0.06) | 0.36 (0.12) | 1.19 | 1.67 | 1.95 | 1.32 |
|  | 75 | 2.55 (0.15) | 1.53 (0.17) | 0.59 (0.27) | 2.57 |  | 0.63 (0.06) | 0.22 (0.06) | 0.33 (0.12) | 0.66 | 4.06 | 7.00 | 1.79 |
| SEATS | 5 | 0.71 (0.03) | 0.36 (0.04) | 0.23 (0.06) | 1.58 |  | 0.78 (0.10) | 0.43 (0.06) | 0.41 (0.19) | 1.04 | 0.87 | 0.85 | 0.58 |
|  | 75 | 1.69 (0.11) | 0.84 (0.21) | 0.58 (0.26) | 1.45 |  | 0.77 (0.04) | 0.41 (0.03) | 0.27 (0.07) | 1.54 | 2.19 | 2.06 | 2.18 |

**Table S3.** Spearman correlation coefficients (r) among prokaryotic parameters and abiotic and biotic factors.

|  | T | S | DIN | SiO_3_^2-^ | PO_4_^3-^ | HNA | LNA | HNF | VLP | HNA% | PPG-H | PMM-H | VMM-H | PPG-L | PMM-L | VMM-L |
| --- | --- | --- | --- | --- | --- | --- | --- | --- | --- | --- | --- | --- | --- | --- | --- | --- |
| T | **—** |  |  |  |  |  |  |  |  |  |  |  |  |  |  |  |
| S | ns | **—** |  |  |  |  |  |  |  |  |  |  |  |  |  |  |
| DIN | -0.85*** | ns | **—** |  |  |  |  |  |  |  |  |  |  |  |  |  |
| SiO_3_^2-^ | -0.83*** | ns | 0.81*** | **—** |  |  |  |  |  |  |  |  |  |  |  |  |
| PO_4_^3-^ | ns | ns | ns | ns | **—** |  |  |  |  |  |  |  |  |  |  |  |
| HNA | -0.53* | ns | ns | 0.44* | ns | **—** |  |  |  |  |  |  |  |  |  |  |
| LNA | ns | ns | ns | ns | ns | 0.73*** | **—** |  |  |  |  |  |  |  |  |  |
| HNF | ns | ns | ns | ns | ns | 0.77** | 0.58* | **—** |  |  |  |  |  |  |  |  |
| VLP | ns | ns | ns | ns | ns | 0.46* | 0.63** | 0.61* | **—** |  |  |  |  |  |  |  |
| HNA% | -0.55* | ns | 0.51* | 0.62** | ns | 0.57* | ns | 0.72* | ns | **—** |  |  |  |  |  |  |
| PPG-H | -0.81** | ns | 0.80** | 0.82** | ns | 0.87*** | ns | 0.71* | ns | 0.86*** | **—** |  |  |  |  |  |
| PMM-H | -0.62* | ns | 0.63* | 0.71* | ns | 0.79** | ns | 0.76** | ns | 0.75** | 0.94*** | **—** |  |  |  |  |
| VMM-H | -0.65* | 0.59* | ns | ns | ns | ns | ns | ns | ns | ns | 0.58* | 0.60* | **—** |  |  |  |
| PPG-L | 0.78* | ns | -0.72* | -0.83** | ns | -0.68* | ns | -0.70* | ns | -0.81** | -0.81** | -0.82** | -0.68* | **—** |  |  |
| PMM-L | 0.87*** | ns | -0.86*** | -0.75** | ns | -0.56* | ns | -0.60* | ns | -0.78** | -0.76** | -0.61* | ns | 0.78** | **—** |  |
| VMM-L | 0.64* | ns | -0.58* | -0.78** | ns | -0.84** | ns | -0.71* | -0.75** | -0.77** | -0.77** | -0.79** | ns | 0.83** | ns | **—** |

ns, nonsignificant. * denotes correlation significant at 0.05 level, ** correlation significant at 0.01 level, and *** correlation significant at 0.001 level.
