# Supplementary material for: Elevated Contribution of Low Nucleic Acid Prokaryotes and Viral Lysis to the Prokaryotic Community Along the Nutrient Gradient From an Estuary to Open Ocean Transect
Source: Front Microbiol. 2020 Dec 15;11:612053. doi: 10.3389/fmicb.2020.612053 (PMC7793805; doi:10.3389/fmicb.2020.612053)
Supplement: Supplementary file 2 [file Data_Sheet_2.pdf]

Supplementary material

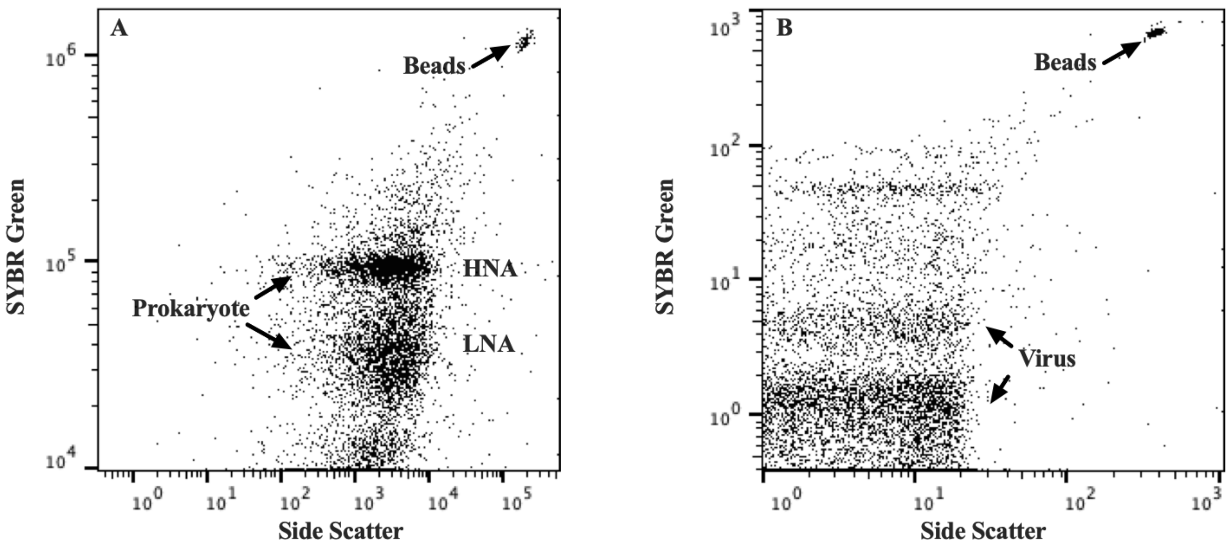

Figure S1. Cytometric plot graph of the (A) prokaryotic and (B) viral populations in samples from SEATS subsurface. The black arrows in panel (A) highlight the prokaryotic cells of high nucleic acid (HNA) and low nucleic acid (LNA) contents and in panel (B) highlight the virus-like particles (VLP).

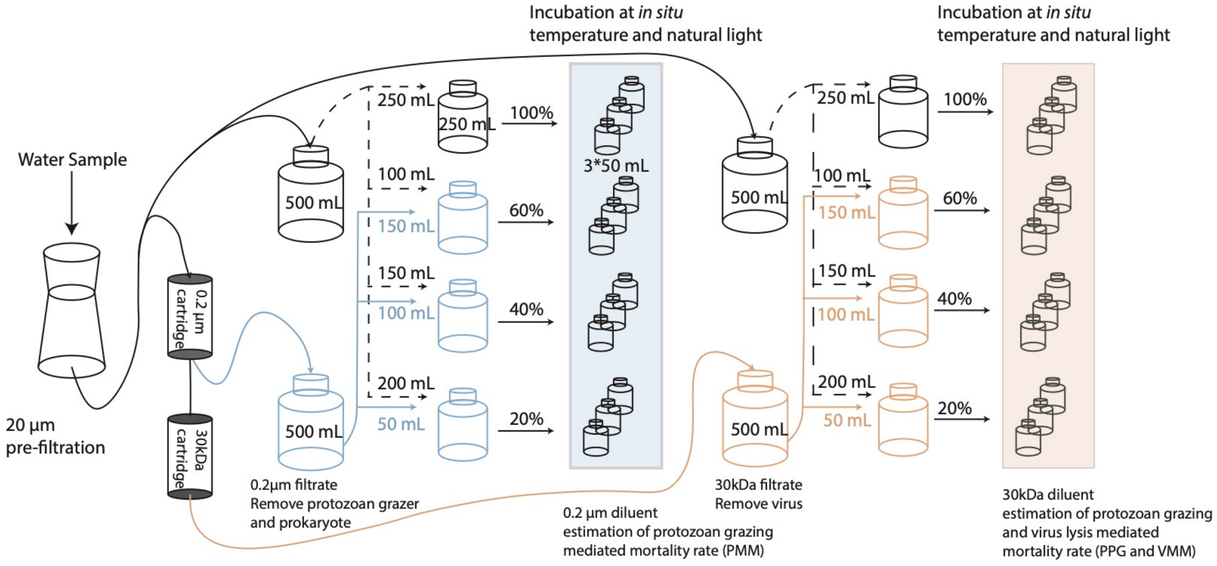

Figure S2. Schematic of procedure of modified dilution experiment to measure potential prokaryotic growth rate and protozoan grazing- and viral lysis-mediated mortality rates based on the tangential flow filtration system.

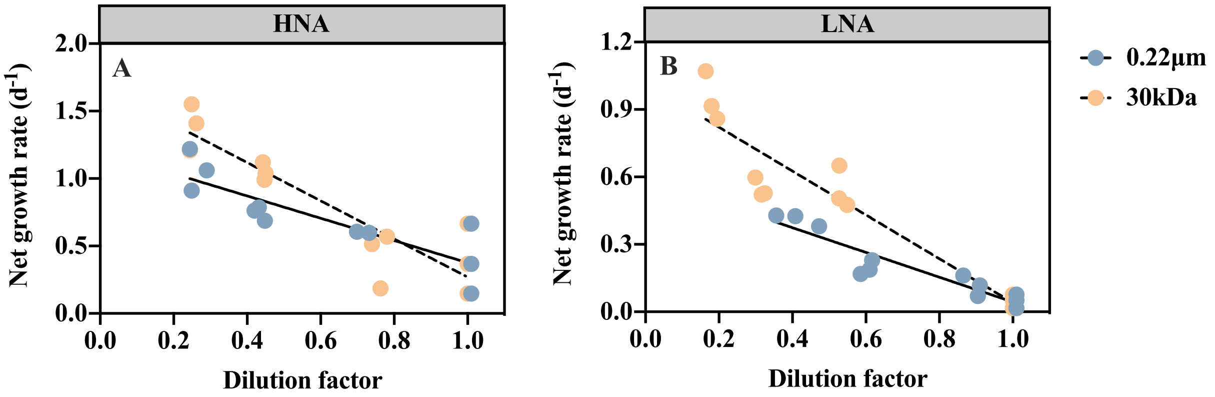

Figure S3. Prokaryotic net growth rate versus fraction of sample water in the dilution series experiments for (A) HNA and (B) LNA prokaryotic groups in samples from subsurface water at SEATS station.

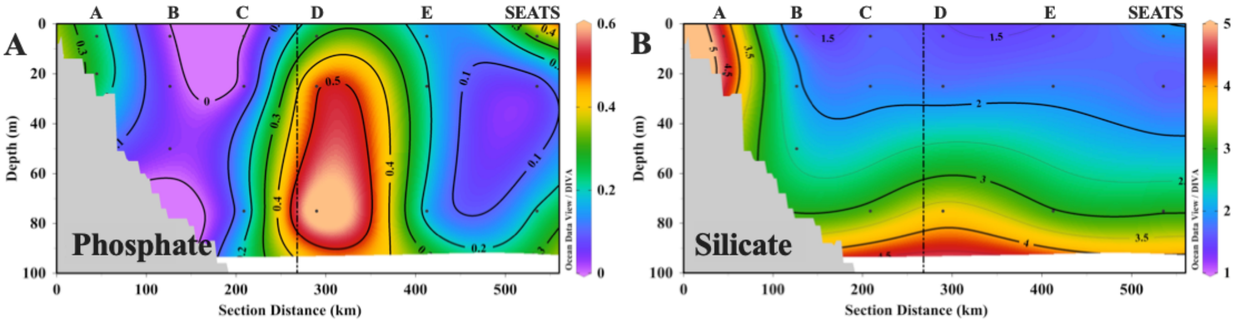

Figure S4. Transect distributions of (A) phosphate concentration (µmol L^{-1}) and (B) silicate concentration (µmol L^{-1}).

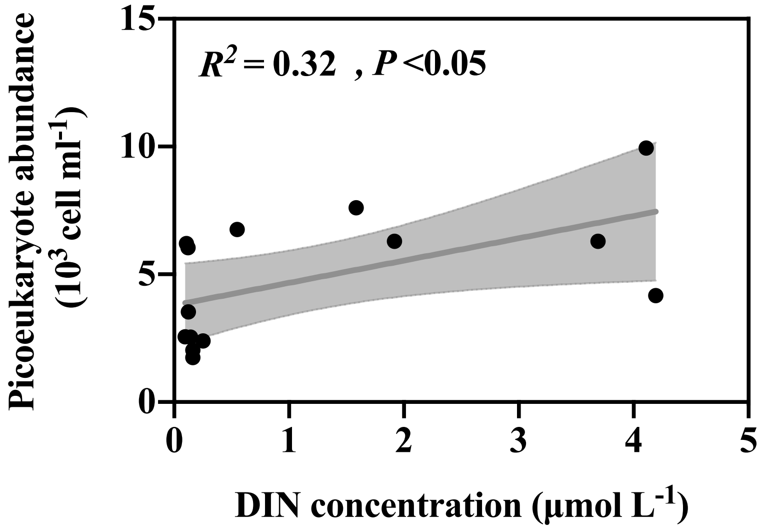

Figure S5. Linear correlations between DIN concentration (μmol L^{-1}) and the picoeukaryote abundance (10^3 cell mL^{-1}).
